# Health and Disease—Emergent States Resulting From Adaptive Social and Biological Network Interactions

**DOI:** 10.3389/fmed.2019.00059

**Published:** 2019-03-28

**Authors:** Joachim P. Sturmberg, Martin Picard, David C. Aron, Jeanette M. Bennett, Johannes Bircher, Mark J. deHaven, Sanne M. W. Gijzel, Henry H. Heng, James A. Marcum, Carmel M. Martin, Andrew Miles, Chris L. Peterson, Nicolas Rohleder, Christine Walker, Marcel G. M. Olde Rikkert, René J. F. Melis

**Affiliations:** ^1^Faculty of Health and Medicine, School of Medicine and Public Health, University of Newcastle, Callaghan, NSW, Australia; ^2^Division of Behavioral Medicine, Department of Psychiatry and Neurology, The H. Houston Merritt Center, Columbia Translational Neuroscience Initiative, Columbia Aging Center, Columbia University Medical Center, Columbia University, New York, NY, United States; ^3^School of Medicine, Weatherhead School of Management, Louis Stokes Cleveland VA Medical Center, Case Western Reserve University, Cleveland, OH, United States; ^4^Department of Psychological Science, University of North Carolina at Charlotte, Charlotte, NC, United States; ^5^Hepatology, Department for Biomedical Research, University of Bern, Bern, Switzerland; ^6^Health and Human Services, College of Health and Human Services, University of North Carolina at Charlotte, Charlotte, NC, United States; ^7^Department Geriatric Medicine, Radboud University Medical Center, Nijmegen, Netherlands; ^8^Department of Pathology, Center for Molecular Medicine and Genetics, School of Medicine, Wayne State University, Detroit, MI, United States; ^9^Philosophy and Medical Humanities, Baylor University, Waco, TX, United States; ^10^Department of Medicine, Nursing and Allied Health, Monash Health, Melbourne, VIC, Australia; ^11^European Society for Person Centered Healthcare, London, United Kingdom; ^12^School of Humanities and Social Sciences, La Trobe University, Bundoora, VIC, Australia; ^13^Friedrich-Alexander-Universität Erlangen-Nürnberg, Erlangen, Germany; ^14^Chronic Illness Alliance, Moonee Ponds, VIC, Australia

**Keywords:** health, top-down and bottom-up causation, disease networks, complex adaptive nature of health, physiology of health, psychoneuroimmunology, health system redesign, emergence

## Abstract

Health is an adaptive state unique to each person. This subjective state must be distinguished from the objective state of disease. The experience of health and illness (or poor health) can occur both in the absence and presence of objective disease. Given that the subjective experience of health, as well as the finding of objective disease in the community, follow a Pareto distribution, the following questions arise: What are the processes that allow the emergence of four observable states—(1) subjective health in the absence of objective disease, (2) subjective health in the presence of objective disease, (3) illness in the absence of objective disease, and (4) illness in the presence of objective disease? If we consider each individual as a unique biological system, these four health states must emerge from physiological network structures and personal behaviors. The underlying physiological mechanisms primarily arise from the dynamics of external environmental and internal patho/physiological stimuli, which activate regulatory systems including the hypothalamic-pituitary-adrenal axis and autonomic nervous system. Together with other systems, they enable feedback interactions between all of the person's system domains and impact on his system's entropy. These interactions affect individual behaviors, emotional, and cognitive responses, as well as molecular, cellular, and organ system level functions. This paper explores the hypothesis that health is an emergent state that arises from hierarchical network interactions between a person's external environment and internal physiology. As a result, the concept of health synthesizes available qualitative and quantitative evidence of interdependencies and constraints that indicate its top-down and bottom-up causative mechanisms. Thus, to provide effective care, we must use strategies that combine person-centeredness with the scientific approaches that address the molecular network physiology, which together underpin health and disease. Moreover, we propose that good health can also be promoted by strengthening resilience and self-efficacy at the personal and social level, and via cohesion at the population level. Understanding health as a state that is both individualized and that emerges from multi-scale interdependencies between microlevel physiological mechanisms of health and disease and macrolevel societal domains may provide the basis for a new public discourse for health service and health system redesign.

*Each culture must provide a more or less successful way of dealing with its environment, both adapting to it and changing it. Moreover, each culture must define a social reality within which people have roles that make sense to them and in terms of which they can function socially. Not surprisingly, the social reality defined by a culture affects its conception of physical reality. What is real for an individual as a member of a culture is a product both of his social reality and of the way in which that shapes his experience of the physical world*.

Lakoff & Johnson—Metaphors we live by (p. 147)

Social and cultural influences distinctly shape people's perception of *health*. In the Western world *health, healthcare*, and the *healthcare system* mostly evoke images of *non-health*, i.e., pictures of personal *suffering* and/or disablement and threat of death from *diseases* like cancer, emphysema or heart disease. Unsurprisingly then the role of the *healthcare system* is seen as that of a *repair shop*.

However, these images are neither congruent with the epidemiology of health in the community (1–3) nor do they reflect the frequency of *clinical disease* detected in primary *health care encounters* (4–6) (Figure 1). Discovering incongruences between perceptions (i.e., mental models) and the physical reality necessitates the search for mental models that better reflect the realities of the *real world* (8–14). In this paper we explore *health, and by implication disease, as an emergent state*. Health, as an emergent state, is the result of the interplay amongst environmental, socio-cultural and economic-political contexts and internal biological potentials, each of which is organized in complex adaptive networks. This understanding of health has implications for health care delivery and health system redesigns.

**Figure 1:**
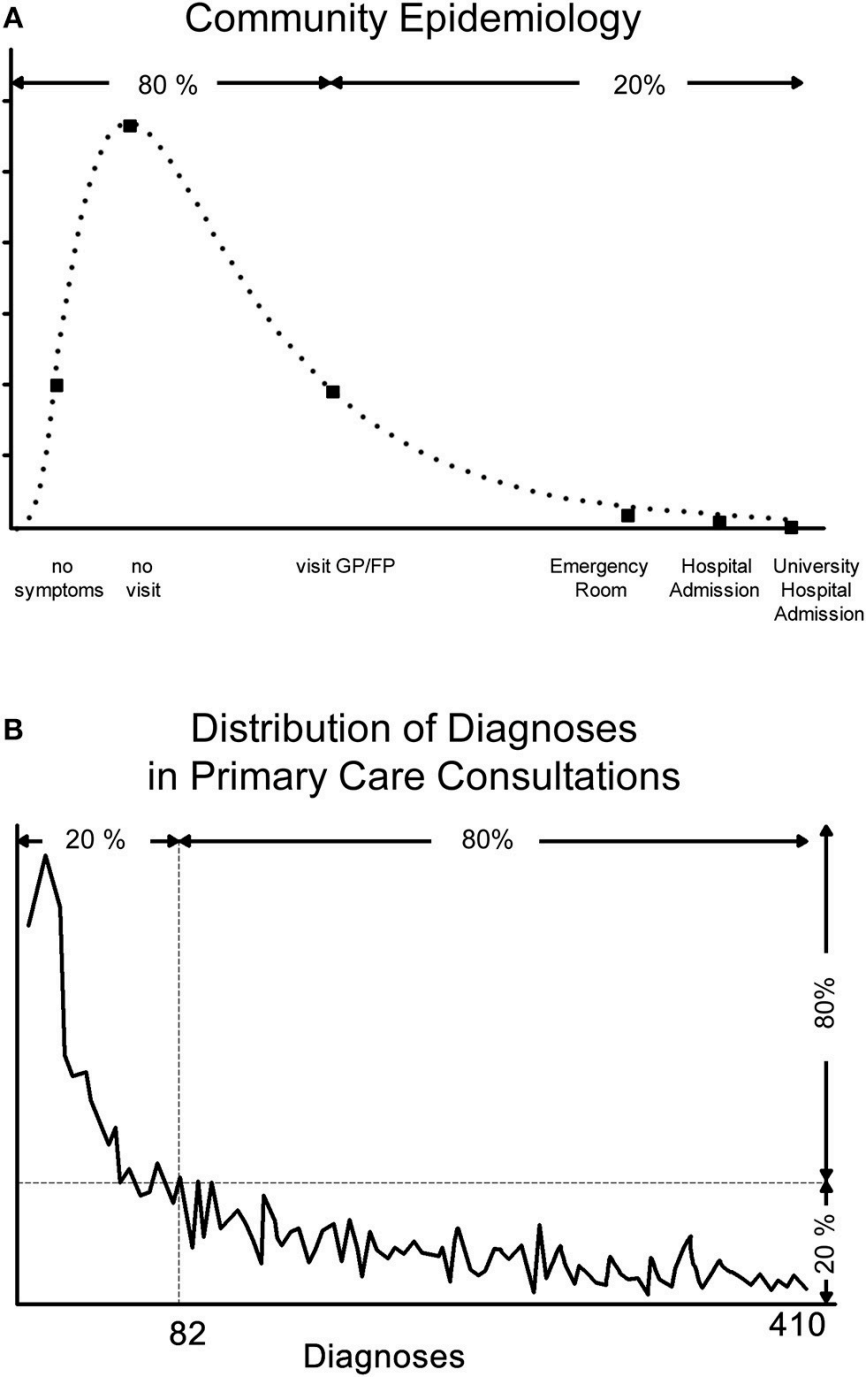
**(A)** Community Epidemiology and **(B)** Diagnoses Distribution Resulting from Primary Care Consultations. The “ecology of medical care” has shown that the Pareto distribution applies to the need for health care−80% of people are healthy or feel healthy enough not to require health care; of the remaining 20%, 80% solely require primary care services (16% of the total), of the remaining 20%, 80% require secondary care (3.2% of the total); and the remaining 20% require tertiary care (0.8% of the total). Vice versa, Braun's studies showed that 80% of all primary consultations result in 20% of all diagnoses (mostly unspecific), the remaining 20% of consultations cover 80% of all diagnoses (4). The key message from these studies is that subjective health/illness experience and objective disease/diagnosis often do not coincide—the majority of people who may have symptoms but no or little illness experience rightly do not report to a care provider, although some will ultimately be found to have an identifiable disease, and many people who seek help because of their degree of illness experience ultimately do not have objectively identifiable disease.

## Toward an Emergent Understanding of Health and Disease

So far no attempt has succeeded to define health in a coherent way (15, 16). Health is a *state of the whole person*, consistent with the word's Old-English etymology of *hal* meaning *whole*. The term *illness* refers to the state of *not being whole*, and needs to be distinguished from *disease* defined by the presence of identifiable pathology or dysfunction (17). We previously explored the notion of health and disease from our respective epistemological, genomic, network physiology, personhood, and social perspectives (18–27) only to find that none of them on their own appeared sufficient to explain the observed *patterns and variations* in distribution of health and disease at both the subjective and objective level.

We suggest that this inability to reduce health to a single construct driven by a single bottom-up mechanism, or a combination of mechanisms, is due to the fact that health is an emergent property of a complex, dynamic, and adaptive system (28). What distinguishes living complex, dynamic and adaptive systems from non-living inert systems is the fact that the behavior of the former are not predictable based on a given set of measurable features. These systems are therefore said to behave *non-linearly* and to exhibit, as a whole, emergent properties that cannot directly be understood based on understanding their individual parts (see Box 1 for explorations of key complexity terms).

Box 1 **Key complex adaptive system concepts.**

**Complex adaptive system**–complex systems whose elements (or agents) learn and adapt their behaviors to changing environments through self-organization, i.e. without external control. Self-organization arises from internal feedback and underpins emergence.

**Dynamic systems**, by their very nature of constant activity, are never in exactly the *same state*. Over time they can emerge into a different state (like a person can be healthy, develop appendicitis and being sick, being in recuperation, before returning back to being healthy), or they may permanently change into a new and different state (like a person being involved in a car accident that makes him an amputee, a paraplegic or a person with a brain injury).

**Emergence**–the ability of individual components of a large system to work together to give rise to unexpected new and diverse behaviors that are not present in or predictable from its individual components.

**Networks/Network Sciences**–study of complex networks of any kind, agents/actors are represented as nodes, their links as edges, network sciences produce predictive models of the behavior of complex networks.

**Non-linearity**–the response to a stimulus is not proportional to its input which can lead to sudden massive and stochastic changes of a system's behavior. Non-linearity is one of the reasons that explain the inherent uncertainties in complex adaptive systems. A typical non-linear distribution curve in biological and social systems is the Pareto distribution, also known as the 80:20 split (see examples in Figure 1).

**Pareto distribution**–also known as the 80:20 split, describes the power-law (non-linear) distribution of observable phenomena in biological, social and other systems.

**Readers interested in the historical developments of systems and complexity sciences** should refer to the historical texts of Ashby, von Bertalanffy, Bak, Capra, Gell-Mann, Hollands, Jansh, Kauffman and Prigogine and Stengers.

### Non-linearity and Emergence in Biological Networks

George Ellis (29) emphasized that “[t]*he basis of complexity consist of modular hierarchical structures, leading to emergent levels of structure and function based on lower level network* [function].” In other words, top-down actions provide contextual constraints that limit the possible bottom-up actions.

These characteristics have also been observed by West (30) who showed that organisms exhibit remarkably simple and systemic scaling laws that describe their complex structures and physiological functions across multiple physical and temporal scales. This limits the rate with which resources can be provided to sustain cellular and organ function (30, 31).

In terms of health, biology provides the common bottom-up blueprint to build anatomical structures and physiological functions, while environmental and socio-cultural top-down constraints limit the emergence of possible health states. Health states, which can be both defined subjectively as health experiences and objectively as taxonomies of disease, thus are not static but rather *dynamic emergent whole person phenomena*.

Dynamic physiological network responses to perturbations provide adaptive *homeostatic dynamics* (32) that allow a person to transition to different *stable health states* across the life trajectory and the inevitable accumulation of diseases and frailty (33–35).

### Emergence of Health (as Well as Disease)

Two features support the view that health is an emergent phenomenon. First, health is inherently related to the interconnected nature of *fractal* anatomy and physiology (30), and second, health is influenced by the layered hierarchical nature of interdependencies between the environmental and socio-cultural environments at the largest scale, personal behaviors at the intermediate scale, and molecular and physiological factors at the smallest scale. Furthermore, health and illness are also subjective states and this additional dimension needs to be distinguished from the objective taxonomic finding of disease. In particular, health can be experienced both in the presence as absence of objective disease. The latter is true exactly for the fact that a human being—as described in the previous paragraphs—is able to adapt to new situations with a *restoration* of experiencing health despite objective loss of function or accumulation of physical, emotional, social, and/or cognitive damage.

**Figure 2:**
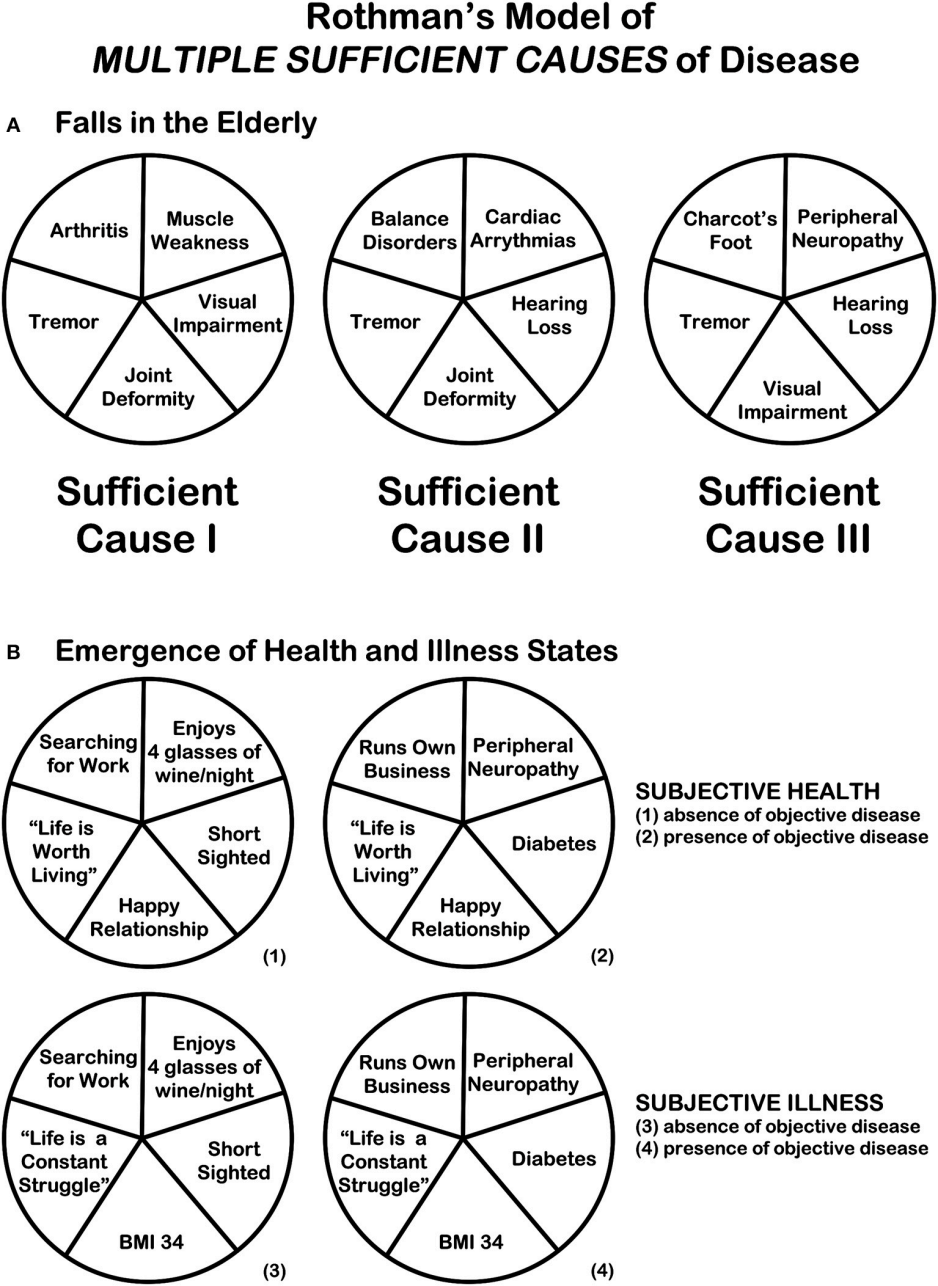
Rothman's model of the “multiple different combinations of sufficient causes” of disease [adapted from Rothman (7)]. **(A)** Rothman's model of the “multiple different combinations of sufficient causes” illustrated in relations to “falls in the elderly.” As Rothman explained: “A specific effect [a fall in the elderly] may result from a variety of different sufficient causes [three causes are illustrated]. The different constellations of component causes [Tremor, Joint Deformity, Visual Impairment, Muscle Weakness, Arthritis, Hearing Loss, Cardiac Arrythmias, Balance Disorders, Peripheral Neuropathy, Charcot's Foot] which produce the effect may or may not have common elements. If there exists a COMPONENT CAUSE which is a member of every SUFFICIENT CAUSE, such a component is termed a *NECESSARY* CAUSE [in this case: only “Tremor” is a necessary cause]. [The f]igure suggests many synergistic relationships. For example, “[Arthritis]” and “[Muscle Weakness]” are completely synergistic with each other and each is partially synergistic with “[Tremor],” “[Joint Deformity],” and “[Visual Impairment].” Partial synergy exists between “[Joint Deformity]” and “[Visual Impairment]”—their effect is dependent on their joint presence in one sufficient cause, but each also has independent effects in another sufficient cause [“Joint Deformity” in Sufficient Cause II, “Visual Impairment” in Sufficient Cause III].” **(B)** Rothman's model can be applied to the EMERGENCE of HEALTH and ILLNESS STATES in the presence and absence of disease. In the example “Life is Worth Living” and “Happy Relationship” are two components necessary for the emergence of the two “HEALTH STATES” (1) and (2), and “Life is a Constant Struggle” and “BMI 34” are two components necessary for the emergence of the two “ILLNESS STATES” (3) and (4).

Rothman's (7) exploration of *multiple different combinations of sufficient causes* resulting or preventing the occurrence of manifest disease supports the non-linearity that underpins the emergent nature of health. Diseases themselves, especially chronic age-related diseases with a multifactorial nature can also be best explained by this component cause model. The model can, however, also be helpful to understand (dis)congruence of illness and disease states (Figure 2). In many situations disease presence and illness experience coincide as diseases are obviously an important *component cause* amongst the many *multiple sufficient causes* that trigger the illness/health experience.

However, we contend that there will also be *sufficient causes* for illness experiences where objective disease is not a *component cause*, i.e., disease presence is not a necessary cause. Vice versa, there may be several *component causes* which *neutralize* the presence of objective disease in their combined impact on the illness experience, i.e., these combinations of factors result in the situation where objective disease is not accompanied by a profound illness experience and thus perhaps—depending on the definition—lack of health.

These introductory remarks expand on Engel's biopsychosocial model of health (36, 37) and build on the implicit and explicit ideas on the *dynamic nature of health* by von Uexküll and Pauli (38), McWhinney (39), Bircher (40), Bircher and Kuruvilla (41), Sturmberg (15, 16), and Huber et al. (42). We propose that

- Health is a *system state* unique to each and every individual arising from the network dynamics of his internal physiological function, external physical and social environments and the ways of making sense of one's experiences.
- Health is also the state that allows one to adapt to changing circumstances and demands that challenge the entropy of the system.

Embracing the theoretical framework of network relationships and dynamic interactions amongst the multitude of different factors impacting on health as a systems state, we describe—in the sense of a hypothesis—how any of four different health states can emerge *clinically* in *seemingly the same* circumstances—(1) subjective health in the absence of objective disease, (2) subjective health in the presence of objective disease, (3) illness in the absence of objective disease, and (4) illness in the presence of objective disease.

We also discuss how health experiences across the life span, socio-economic strata, and cultural norms reflect a human's adaptive capacities.

The dynamic adaptive behavior of the networks of a complex adaptive system results in variable and non-predictive outcomes—here four different health states—and thus challenge the widely held belief of strict linear causal chains resulting in particular health outcomes.

## Health—a Whole Person State

**Figure 3:**
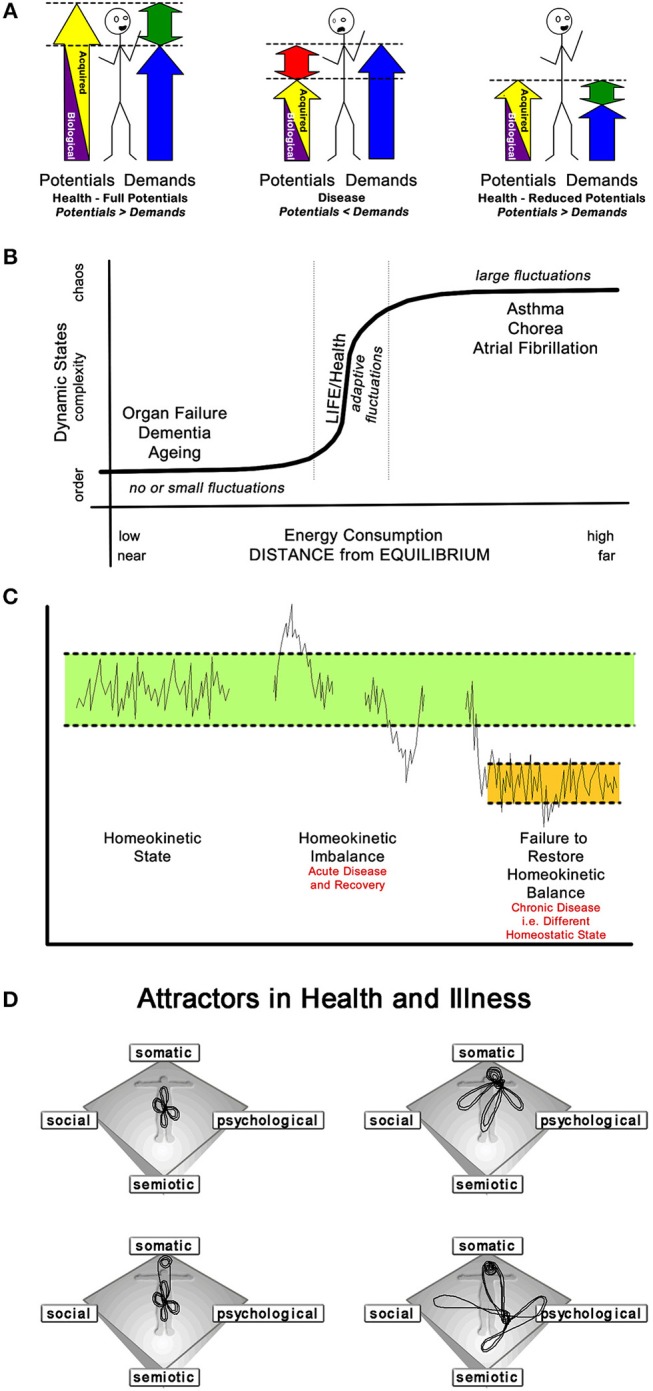
Four “Mutually Agreeable” Conceptualisations of Health and Disease. **(A)** Bircher's model defines health as an im/balance between the two acquired potentials and the demands of life *(biologically given and personally acquired resources)*. *The relationship between the total potentials and the demands of life determines, whether an individual is in a health* (potentials > demands or demands < potentials) or *disease* (demands > potentials) state. Note: Health can be achieved despite a reduced level of the potentials (40). **(B)** Macklem describes life/health as a transition phenomenon. His [s]*chematic illustrate*[s] *the continuum of open thermodynamic systems from ordered, near-to-chaotic*, [to] *far-from-equilibrium states. As energy consumption increases, systems move further from equilibrium and pass through a phase transition between order and deterministic chaos. Complex systems, like life* [and health]*, exist in this phase transition* (44). **(C)** Soodak pointed to *Bernard's principle of the constancy of the internal environment (that is, homeostatic regulation, which may be both cyclic and adaptive) as the condition of free and independent life, is the first approximation to a theory of the organism*. He suggested *the new physical doctrine of homeokinesis as a second proximation to such a theory of complex autonomic systems*. Recognizing the *natural hierarchy of organizational levels* allows *a dynamic regulation scheme* that allows the *homeostatic persistence* [to be] *maintained by the action of chains of thermodynamic engine processes*, [i.e., physiological interactions across all scales of human existence] (32). **(D)** Health, as an attractor, maintains a dynamic equilibrium over time. Thus, *health cannot be “one” particular state, but rather a relative point in a phase space, i.e., health is a chaotic attractor. Three attractor patterns emerge; a health attractor around the center of the somato-psycho-socio-semiotic phase space, with illness, dis-ease, and [acute self-limiting] disease occur on “more distant orbits” of the attractor; a chronic disease attractor—*here—*centered toward the somatic corner of the phase space* (but can be any other corner)*; and a “psychosomatic attractor” whose dynamics swap between two phase space areas, the physical and the psycho-semiotic* (16).

Some degree of health is a prerequisite for life, and life—like health—occurs in an open thermodynamic system at the transition between an ordered stable and a chaotic disordered state (43–45) (Figure 3). The maintenance of health therefore necessarily entails constant energy flux through the system (46). Health as the state of *wholeness* in the narrow discontinuous window between these two thermodynamic states requires the ability:

- To have the required resources to meet and cope with the demands of life
- To respond to stressors arising in one's environment
- To maintain one's internal homeokinetic balance
- To adapt to losses in resources, stressors, and homeostatic instability
- To manage the gap between one's biological potential and life's demands.

These requirements entail that the *state of health* can only be achieved through continual dynamic responses, often non-linear, to all forms of challenges—biological, social, emotional, and/or cognitive—to the *person as a whole* (15, 16, 33).

## Health, Illness, and Disease—the Dynamics of *Whole Person Adaptation*

Personal health/illness experiences and personal diseases cannot be separated from each other—they are a *single whole person phenomenon*. This is most evident looking at the impact of age and disability, neither precludes the *experience of health*. For example, two thirds of the eldest people (85+ years) (47) as well as people with disabilities (48) still rate their health as good or better despite having an increasing number of *disease labels*.

While particular socio-cultural and environmental contexts constrain a person's system, recursive bottom-up physiological actions aim to control the cellular and organ functions that allow the person's system to emerge toward the experiential states of health and non-health (illness as the subjective experience, disease as the objective reason).

### The Physiological System's Component

Mounting evidence indicates the prevailing linear reductive notion that diseases are caused by a single identifiable *cause* is no longer sustainable (12, 49). Rather, diseases emerge as a consequence of interactions among multiple physiological networks—in particular those that regulate gene networks (27, 50), activities of the autonomic nervous system (51) and the hypothalamic-pituitary-adrenal axis (HPA) (52, 53), as well as the bioenergetics within mitochondria in concert with other metabolic pathways (54, 55). These physiological networks are sensitive to changes in psychosocial and environmental parameters and thus may—through positive and/or negative feedback—either enhance health or contribute to the emergence of disease (see below).

#### The Role of the Genome

While genes as individual units provide the necessary information to produce the biological building blocks of cells and the organism, it is the genome, i.e., the gene network interactions, that encodes the *system as a whole* (27).

Recent evidence indicates that common and complex disease is rarely caused by specific gene mutations but rather by genome instability that manifest at the level of DNA methylation and changes in gene expression (27). The external environmental as well as internal physiological perturbations produced by stochastic or random genomic changes (27), rather than *punctuated* common genetic DNA mutations, are responsible for most diseases and their intra-person genome heterogeneity (27, 56, 57). Moreover, different cells may contain unique acquired genetic features in DNA sequence, DNA methylation, and protein expression (58, 59). These multiple cellular variants are essential for cellular adaptation during dynamic environmental change, but as a trade-off, they also contribute to disease (60).

Indeed, the links between the genome networks and the *phenotypic disease* networks, have been defined as the *diseasome* (50). The diseasome shows important genome-linked diseases and clarifies how and why certain diseases occur in clusters within the same person (50, 61).

#### The Role of the Autonomic Nervous System and Hypothalamic-Pituitary-Adrenal Axis Network

Selye (62) initially recognized that *irritating substances* initiate an immune response mediated by HPA-axis activation. He later recognized that external factors can also trigger the same immune response and influence health. He called these factors *stressors*, regardless of them being experienced in a positive or negative way. In addition, Porges demonstrated the integration of the multiple pathways of the vagal system in relation to stressors on organ function (polyvagal theory), visceral regulation, and emotional responsiveness (63, 64).

*Stressors* influence cellular function by modulating gene expression. The release of primary neuropeptides, neurohormones, and neurotransmitters leads to the production of hormones and cytokines (53), which influence the proteomic and metabolic network pathways. When dysregulated or perturbed beyond the adaptive capacity of the system, stressors may ultimately result in the emergence of diseases (65).

At the experiential level, the brain incorporates the perception and appraisal of the current environmental experience (66). If an individual perceives greater coping ability (e.g., more resources or skills) than the situation demands, the body will likely mount an appropriate physiological response, meaning activation with quick recovery. However, if an individual decides their ability to cope whether conscious or subconscious is smaller than the demands of the situation, the interpretation leads to a loss of control—the importance of which has been highlighted by Antonovsky (67, 68)—or threat to self. This perceived threat may cause excessive activation of the stress systems and exaggerated parasympathetic withdrawal.

In a well-regulated body, sympathetic activation leads to elevated epinephrine/norepinephrine, promoting immune activity, in particular proinflammatory cytokine production (69–71). Following the stressor, unused bioavailable cortisol shuts of the neuroendocrine pathway as well as inhibits immune activity, in concert with increased parasympathetic activity (i.e., acetylcholine) as both can inhibit pro-inflammatory gene expression. Thus, during recovery, a well-regulated body critically restores the balance between the neuroendocrine and immune systems.

However, when a chronic stressor is perceived (72), this physiological recovery may not occur. Chronically elevated cortisol can lead to immune cells that become insensitive to cortisol (73). Hence, chronic stress can lead to the removal/reduction of both anti-inflammatory pathways, fuelling proinflammatory cytokine production that stimulates the stress systems—potentially creating a never-ending negative feedback cycle and multi-system dysregulation.

#### The Role of the Metabolic Networks

The mitochondrion regulates energy production as well as intracellular signaling and may be especially sensitive to the effects of elevated cortisol due to chronic stress (74). Mitochondria's morphology and function are altered by neuroendocrine mediators and metabolic changes associated with the stress response. If persistent, mitochondrial damage may lead to mitochondrial allostatic load (MAL) (55). MAL can trigger signaling cascades known to reduce energy production and overall capacity within the cell and influencing cellular gene expression as well as initiate extracellular damage by promoting pathogenic inflammation and altering the circulating metabolome (75). These changes yield broad ranging effects on cell-specific parameters (intracellularly) and whole organism function (systemically). Thus, mitochondria, by providing energy to animate and regulate these different regulatory networks, and via their role in cell and whole-body signaling (46) play a key role in the development of pathological changes across organ systems (54).

Mitochondrial dysfunction can cause organ-specific, as well as multi-systemic defects throughout the organism by increasing oxidative stress (54, 76). Symptoms of mitochondrial disorders often manifest simultaneously in the neuromuscular system causing exercise intolerance and myopathy, in the brain with stroke-like episodes and structural cortical and sub-cortical anomalies, gut dysmotility and constipation, hearing and vision loss, and insulin resistance, among others (77, 78). Likewise, psychological stress and other psychosocial experiences may influence mitochondrial function via multiple neuroendocrine and metabolic mechanisms (74, 79), which influence neuroendocrine, metabolic, and transcriptional responses to acute stress (80). Therefore, metabolic activities within mitochondria may regulate cellular and organismal responses to environmental perturbations and thus contribute to generate individualized states of health or illness.

### Responses to Stressors Is the Key to Understanding Health, Illness, and Disease

The amount of *stressors* has been defined as the quantity and severity of stressful events or stressors that contribute to an overall *allostatic load*. While short-term stressors promote adaptation in a constantly changing environment, persistent, and/or high levels of stressors contribute to enduring physiological dysregulation via neuroendocrine, autonomic, immune, and metabolic mediators (81). These perturbations result in epigenetic changes at the cellular level (82) and result in the accumulation of CNS or organ damage and ultimately leading to the emergence of phenotypic disease and increased premature mortality (26, 83).

#### Network Inter-organ Interactions—The Next Larger Spatial Scale

At the organ level, network interactions between different organs determine the phenotypes of health and disease (31). Indeed, the HPA axis is credited a central neuroendocrine role, as it has been shown to have key interface functions between the biological and psychosocial domains.

Moreover, the HPA axis is also designed to balance pro- and anti-inflammatory activities, and is the principle mediator and regulator between internal and external system perturbations (84, 85). In addition, there is cross-talk between the immune system and the brain; peripheral cytokines can stimulate the HPA axis (86), and induce illness behavior (87, 88) via indirect [e.g., the vagus nerve (63)] or direct (e.g., crossing leaky portions of the blood brain barrier) pathways (89, 90).

Considering the role of the HPA axis in inter-organ interactions within a network science framework, its *edges* and *nodes* have different values and directions. Because of these HPA-edges, CNS activation and cortisol—the principle output of the HPA axis—have the potential to influence almost every cell in the body, making them an integral feature in linking multiple physiological systems. While dysfunction in one organ system can stimulate the CNS and HPA axis and thus trigger dysfunctions in other organ systems, CNS and HPA axis dysfunctions themselves can affect peripheral bodily systems, with the consequence of “creating disease.”

Therefore, the HPA axis may play a special role in limiting or increasing the vulnerability of different organs, similar to those describing the cascading failures in an electricity network (91). However, other organs may also play a similar pivotal role in the human organ network. The vascular tree is a space-filling and volume preserving structure; its fractal nature allows even blood flow across the network and connects organ systems (30). Its vascular edges supply organs with oxygen, nutrients and water, clear metabolic breakdown products, and signal consequences of distress in one part of the system across the entire network (e.g., by hormone transport) (31).

This network understanding of inter-organ interactions has resulted in empirical studies to support the complex adaptive network models that link human biology and psychosocial environment in health and disease (12).

#### The Environmental Impacts on Health and Disease—The Macro Scale

For the external—physical as well as socio-cultural and political—environment to affect health and disease over the life course (92), it has to cause perturbations of the internal physiology (29). The health effects of the external environment result from dysregulated neuroendocrine and CNS network functions as described, and if persistent have epigenetic consequences through gene regulatory effects as described by the emerging field of *social genomics* (53, 93, 94).

These fields of study provide the physiological rational to understand the overwhelming epidemiological evidence of *the macrolevel constraining* influences of socio-economic status on health (92). Poor education, housing, work conditions or underemployment, low income, social segregation, and racial discrimination, frequently coupled with personal lifestyle risk factors such as tobacco use, poor nutrition and lack of physical activity, dysregulate immunoregulation resulting in increased proinflammatory activity leading to disease formation and poor health (95–99).

## Health States Emerge from Integrated Network Interactions

**Figure 4:**
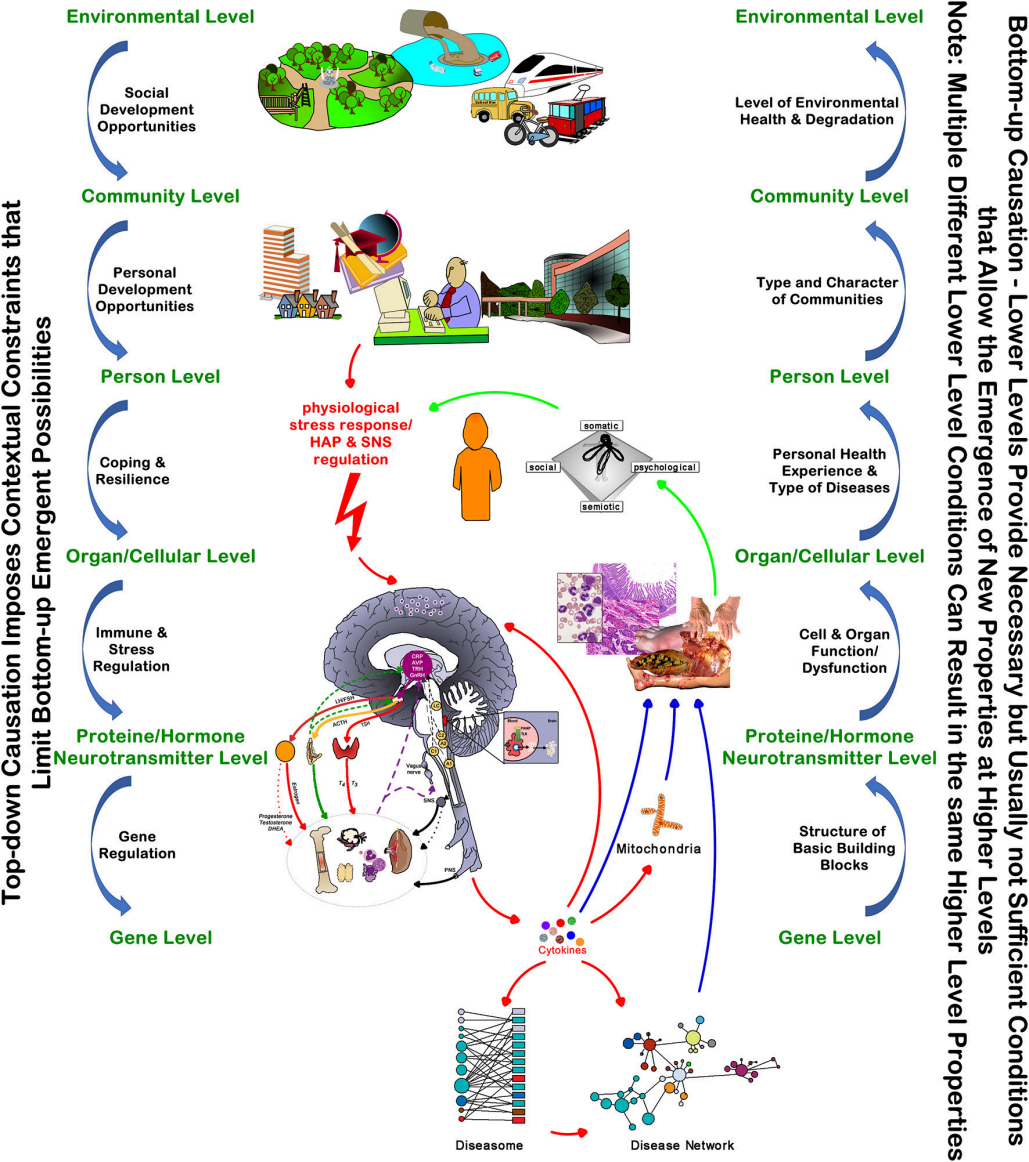
The Top-Down and Bottom-Up Interdependencies of Health and Disease. The model highlights the key “networked” relationships between the external factors and internal mechanisms of the person's health and illness experiences. 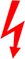 exaggerating effect.

Each of the four health states—(1) subjective health in the absence of objective disease, (2) subjective health in the presence of objective disease, (3) illness in the absence of objective disease, and (4) illness in the presence of objective disease—emerges from integrated network interactions *between* and *across* the multiple scales of structure and function over time (a non-deterministic outcome). As all of these networks are interconnected within a complex adaptive system, they influence each other. This integrated network understanding explains the observations that even minor differences in environments, perceptions or biological function can lead to adaptations of health and/or illness experiences, and that they often overlap with the objective findings of disease/s (Figure 4). At a clinical level the degree of a person's *whole of system adaption* in light of these network dynamics is *clinically* measurable subjectively as *self-rated health* (35, 100–103) and objectively e.g., as *heart rate variability (HRV)* (104, 105).

## Consequences for Health Care^1^ Delivery and Health Care Service Design

The hierarchical modular nature of complex adaptive systems entails that every higher level provides the context, and thus constraint, for the behavior of its lower level components. Health is the *observable state* of top-down and bottom-up network interactions of a person's system components—from the small to the large-scale subsystems, from molecules to man, and beyond man to the socio-cultural and political context of his life. It is these relationships and interactions that make health a *whole person state*. Health, like the occurrence of disease, are emergent within the person but involve different system pathways and dynamics—it is the task of the health professional to untangle the pathways and dynamics for this *particular* patient.

If we want to achieve the best possible health for our patients and our communities we need to have the person and his health/illness experiences—regardless of the concomitant absence or presence of disease—at the center of the healthcare system (Figure 4) (106–110). Importantly, we must appreciate the importance of the constraining environmental and socio-cultural and political level contexts on the adaptive physiological mechanisms underpinning cellular and organ functions. Changing the context in which a system operates often shows greater effects than altering the way the fundamentals operate within the system. These insights have important implications for managing people/patients in the clinical encounter, as well as for all policies and their impact on health and the healthcare system at large (110).

### Focusing Simultaneously on the Patient's Health Experience and His Disease

An understanding of the emergent dynamics with the “person as a system,” being constraint by his environment that limits the emergent possibilities of his biological function, demand an initial focus on the person's evaluation of their health [Antonovsky's salutogenic model (111)]. Pragmatically, asking: “*What are your goals for your care, and how can I help you?*” has been shown to open up this most important conversation (112), and to reveal the underlying dynamics resulting in his current health/illness state.

Managing diseases, mostly regarded as the essence of medical care, ought to be guided by the patient's goals and aspirations—not all of a person's diseases need every available biomedical intervention, and some interventions required to achieve a person's goals and aspirations will be out of the scope of the *traditional medical model* of patient care.

The context of care matters, as the epidemiology of health, illness, and disease have shown, most patients presenting to health professionals do not have acute life-threatening diseases. Those diseases are the ones that invariably benefit, in the short term, from the *traditional disease care model* interventions. The majority of people seeking health care, including those having recovered form a life-threatening disease, require care that explores the network relationships resulting in the illness experience, and a *tweaking* of the *most influential* network nodes that constitute the person's *system as a whole*.

In other words, optimal health outcomes—at the subjective and objective levels—are most likely to be achieved if the health state of the person is understood as an emergent property of their entire system, and if biomedical and psychosocial interventions go hand in hand. George Engel first outlined a system's focused approach to patient care (36, 37); however, three major obstacles impede this approach.

- First, the health system design, in educational, organizational as well as cultural terms, persists as an assembly of dependent units focused in a reductionist way on delivering care for acute problems or single diseases, unable to deal with complexities arising from the chronic sequelae of multiple co-occurring conditions.
- Secondly, medical practices are largely not designed to provide a *network for care*, and
- Thirdly most health systems fail to address the inequalities produced by both social inequalities (including political ideology and government policy) as well as those induced by the health financing system.

### The Need for Health System Redesign

Understanding health/illness and disease as emergent network interaction presenting in one of four health states—(1) subjective health in the absence of objective disease, (2) subjective health in the presence of objective disease, (3) illness in the absence of objective disease, and (4) illness in the presence of objective disease—is a prerequisite for healthcare system improvement. These appreciations shift the redesign focus on three main issues.

- At the macro level, it must focus on the interconnected nature of the social, cultural, and economic-political factors influencing health and disease as the socio-cultural will become biological over the life course (92). In addition, the socioeconomic context has epigenetic effects that have an impact on the health of the next generations and is most detrimental for the most vulnerable portion of the population (113–116).
- At the meso level, it needs to create community partnerships to address disease promoting environmental factors.
- And, at the micro level, we must overcome the single organ focus and embrace the interconnected physiological networks of health and disease (110).

We need organizational redesign of health care services and healthcare systems to link health care professional interventions with personalized social support services and culturally sensitive community-oriented improvements of the physical social and economic infrastructure in local communities. This approach recognizes that health is a social outcome resulting from systematically combining clinical science, collective responsibility, and informed social action (117).

It also must address community priorities in partnership with community members, building on existing community assets while acknowledging the role of cultural factors, and providing evidence that can be used to mobilize and advocate for policies directed at reducing disease risk (118).

At the research level *health system redesign* needs to be supported by a shift in research priorities and funding (119, 120)—from pathogenesis to salutogenesis (120), from improving disease care to improving health care, health maintenance, health promotion and self-care (119), and from biomedical disease eradication to behavioral and social sciences approaches to disease prevention (99, 119).

### How Might It Be Achieved—Suggestions to Start a Broad-Based Discourse

In practical terms, we require a shift toward a dynamic system appreciation of health at the individual and population level (110). To aid this conversion, a critical step will be to begin seeing individuals and their health behaviors as the product of their negotiations and adaptions needed to survive in their life circumstances rather than laziness or lack of concern about their health/illness identity, i.e., exploring the pathways and dynamics leading to this person's health/illness state. This transition will require a modification in training of health and other service providers toward care delivery that integrates macrolevel (mainly environmental, socio-cultural, and political) to microlevel (mainly biological) elements in a person-centered way.

Empirical research at the nexus of hospital, community, primary, and social care indicates many challenges related to roles, responsibilities, and resources across public and private sectors. Ultimately, this is political—as Barbara Starfield stated: *primary healthcare is a “health equity-producing” social policy* (121).

## Conclusions

A complex systems approach understands health and illness as the subjective and disease as the objective emergent states of the top-down and bottom-up interactions between the constraining environmental, socio-cultural and economic-political contexts and the recursive physiological and psychological interactions of cellular and organ function networks.

- The environmental, socio-cultural, and economic-political structures in which a person lives creates the contextual constraints for his internal physiology to realize itself.
- It is the degree of system stress and the inability of the organism to cope adequately, rather than the specific nature (environmental, socio-cultural, economic, political, metabolic) of the stress (122) that drives the overall system toward entropy and causes long-term cellular damage resulting in disease (27).
- The adaptive capacity of the organism and of the person is determined by interconnected energy-dependent biological, physiological, and social network processes that could be targeted to promote health.
- The physiological stress response loop relationship between CNS and HPA axis affects physical disease as much as emotional/cognitive functioning (26) and thus the nature of the socio-cultural and economic-political environment.
- Health and disease causality can be understood best in an adaptive system dynamics framework and in terms of *the life of the human organism* (as a biological object) and *the type of life a human organism is living* (as a person) (117).
- The etiology of both disease states and illness experiences can be best explained and visualized with the component (necessary and sufficient) cause model as developed by Rothman (7).
- The state of the physiological network integration between the physical, socio-cultural, economic, political, emotional, and cognitive domains (123–125) as well as the therapeutic effectiveness (126) can be assessed subjectively elucidating the state of self-rated health, and objectively by monitoring heart rate variability (HRV).

The outlined dynamics between the environmental/socio-cultural/economic-political/emotional-cognitive and biological domains explain the emergence of four observable patterns of health states—(1) subjective health in the absence of objective disease, (2) subjective health in the presence of objective disease, (3) illness in the absence of objective disease, and (4) illness in the presence of objective disease.

Healthcare providers must recognize that people live in socio-cultural, political, and economic systems that shape behaviors and influence access to the resources they need to maintain good health (97). Clinical management needs to be coordinated—ideally in a face-to-face manner—amongst all relevant providers (24) and to take into account the persons capacity to handle treatment regimens and self-management strategies (20, 127). The focus of treatment should be amelioration of and improved coping with stressors as a major driver for poor subjective health and objective disease (128).

In conclusion, this paper attempted a synthesis of facts and theories about health and disease that result in the four observable health states. It led to the insight that the effective care of patients' illnesses requires a strategy that combines person-centeredness with the scientific approach of managing the molecular network physiology, which together underlie the emergence of subjective health experiences and objective disease formation. This approach results in strengthening resilience and self-efficacy (129), builds social capital and cohesion (97, 110), and offers some pragmatic suggestions to start a necessary broad based discourse to shape the future directions of medical and social care.

Finally, we suggest that ideas explored in this paper should be tested pragmatically in routine clinical practice to capture the individualized state of a person's health and to better understand and treat his illness.

- At the most basic level the routine integration of asking the patient to “self-rate” their health—no different to routinely checking their vital signs—not only would provide important information about the current state of health/illness experience, but also guide health professionals in managing the *most appropriate (biological, social, emotional, or cognitive) domain* underlying the patient's illness.
- HRV monitors are becoming readily available and may provide a simple objective measure to *quantify the overall physiological state* of a person's system function, however, its validity in non-critical clinical practice will require further study.
- Ongoing research aims to provide clinicians with other objective measures of the functional state of regulatory systems, including mitochondrial bioenergetics, HPA axis function, inflammatory load, and autonomic regulation.
- It is, however, important to highlight that *very similar adaptive states* can be achieved by very different combinations of objective measures. This awareness should drive future research endeavors into understanding the nature of health, illness, and disease as the basis for best possible health care delivery and health system design.

## Author Contributions

JS developed the ideas for this paper, and RM provided substantial inputs into its refinement. JS and RM are the guarantors for this paper. JS wrote the first draft, and all other authors contributed to the synthesis and revisions of the paper. All authors approve the final version of the article.

### Conflict of Interest Statement

The authors declare that the research was conducted in the absence of any commercial or financial relationships that could be construed as a potential conflict of interest.

## Footnotes

^1^*Health care* refers to the “delivery of care”, *healthcare*- as in healthcare system - means the “organizational institution.”

## References

[1] White K Williams F Greenberg B. The ecology of medical care. N Engl J Med. (1961) 265:885–92. 10.1056/NEJM196111022651805 14006536

[2] Green L Fryer G Yawn B Lanier D Dovey S. The ecology of medical care revisited. N Engl J Med. (2001) 344:2021–5. 10.1056/NEJM200106283442611 11430334

[3] Johansen ME Kircher SM Huerta TR. Reexamining the ecology of medical care. N Engl J Med. (2016) 374:495–6. 10.1056/NEJMc1506109 26840150

[4] Braun RN Die gezielte Diagnostik in der Praxis. Stuttgart: Schattauer (1957).

[5] Aitken AM Braun RN Fraillon JMG Understanding General Practice. Melbourne: The Victorian Academy for General Practice (1982).

[6] Fink W Kipatov V Konitzer M Diagnoses by general practitioners: accuracy and reliability. Int J Forecast. (2009) 25:784–93. 10.1016/j.ijforecast.2009.05.023

[7] Rothman KJ. Causes. Am J Epidemiol. (1976) 104:587–92. 10.1093/oxfordjournals.aje.a112335 998606

[8] Box GEP Draper NR Empirical Model Building and Response Surfaces. New York, NY: John Wiley & Sons (1987).

[9] Mikulecky DC Reductionism and Complexity: Continum or Dichotomy? (2005). Available online at: http://www.people.vcu.edu/m~ikuleck/oct2005lec.PPT (Accessed 04-03-2006).

[10] Snowden DJ. Multi-ontology sense making: a new simplicity in decision making. Inform Primary Care. (2005) 13:45–53. 10.14236/jhi.v13i1.578 15949175

[11] Pidd M Tools for Thinking: Modelling in Management Science. Chichester: John Wiley & Sons (2010).

[12] Greene JA Loscalzo J. Putting the patient back together — social medicine, network medicine, and the limits of reductionism. N Engl J Med. (2017) 377:2493–9. 10.1056/NEJMms1706744 29262277

[13] Kahneman D Thinking, Fast and Slow, Farrar. New York, NY: Straus and Giroux (2011).

[14] Gigerenzer G Smart Heuristics. In: Brockman J editor. Thinking: The New Science of Decision-Making, Problem-Solving, and Prediction. New York, NY: Harper Perennial (2013), p. 39–54.

[15] Sturmberg JP. The personal nature of health. J Eval Clin Pract. (2009) 15:766–9. 10.1111/j.1365-2753.2009.01225.x 19674233

[16] Sturmberg JP Health: a personal complex-adaptive state. In: Sturmberg JP Martin CM editors. Handbook of Systems and Complexity in Health. New York, NY: Springer (2013), p. 231–42.

[17] Boorse C Health as a theoretical concept. Philos Sci. (1977) 44:542–73. 10.1086/288768

[18] Sturmberg JP Bennett JM Martin CM Picard M. ‘Multimorbidity’ as the manifestation of network disturbances. J Eval Clin Pract. (2017) 23:199–208. 10.1111/jep.12587 27421249

[19] Miles A Asbridge JE. Multimorbidity—a manifestation of network disturbances? How to investigate? How to treat? J Eval Clin Pract. (2017) 23:193–8. 10.1111/jep.12723 28239933

[20] Walker C Peterson CL. Multimorbidity: a sociological perspective of systems. J Eval Clin Pract. (2017) 23:209–12. 10.1111/jep.12599 27440439

[21] Marcum JA. Multimorbidity, P4 medicine and holism. J Eval Clin Pract. (2017) 23:213–5. 10.1111/jep.12588 27357479

[22] Melis RJF Gijzel SMW Olde Rikkert MGM. Moving beyond multimorbidity as a simple count of diseases. J Eval Clin Pract. (2017) 23:216–8. 10.1111/jep.12693 28052469

[23] DeHaven MJ. Multimorbidity, chronic disease, and community health science. J Eval Clin Pract. (2017) 23:219–21. 10.1111/jep.12632 27569572

[24] Bircher J Hahn EG. “Multimorbidity” as the manifestation of network disturbances. From nosology to the Meikirch model. J Eval Clin Pract. (2017) 23:222–4. 10.1111/jep.12633 27619725

[25] Aron DC. Multimorbidity: an endocrinologist looks at multi-level network disruption and at what gets diabetes? J Eval Clin Pract. (2017) 23:225–9. 10.1111/jep.12600 27440485

[26] Rohleder N Translating biobehavioral research advances into improvements in health care—a “network of networks” approach to multimorbidity. J Eval Clin Pract. (2017) 23:230–2. 10.1111/jep.12657 27747965

[27] Heng HH. Heterogeneity-mediated cellular adaptation and its trade-off: searching for the general principles of diseases. J Eval Clin Pract. (2017) 23:233–7. 10.1111/jep.12598 27421676

[28] Olde Rikkert MGM Dakos V Buchman TG Glass L Cramer AOJ Levin S. Slowing down of recovery as generic risk marker for acute severity transitions in chronic diseases. Crit Care Med. (2016) 44:601–6. 10.1097/CCM.0000000000001564 26765499

[29] Ellis GFR. Top-down causation and emergence: some comments on mechanisms. Interface Focus. (2012) 2:126–40. 10.1098/rsfs.2011.0062 23386967 PMC3262299

[30] West GB The origin of universal scaling laws in biology. Phys A. (1999) 263:104–13. 10.1016/S0378-4371(98)00639-6

[31] Kriete A Sokhansanj BA Coppock DL West GB. Systems approaches to the networks of aging. Ageing Res Rev. (2006) 5:434–48. 10.1016/j.arr.2006.06.002 16904954

[32] Soodak H Iberall A. Homeokinetics: a physical science for complex systems. Science. (1978) 201:579–82. 10.1126/science.201.4356.579 17794110

[33] Yates FE Homeokinetics/homeodynamics: a physical heuristic for life and complexity. Ecol Psychol. (2008) 20:148–79. 10.1080/10407410801977546

[34] Ferrucci L Giallauria F Schlessinger D. Mapping the road to resilience: novel math for the study of frailty. Mech Ageing Dev. (2008) 129:677–9. 10.1016/j.mad.2008.09.007 18929593 PMC2630702

[35] Gijzel SMW van de Leemput IA Scheffer M Roppolo M Olde Rikkert MGM Melis RJF. Dynamical resilience indicators in time series of self-rated health correspond to frailty levels in older adults. J Gerontol Ser A. (2017) 72:991–6. 10.1093/gerona/glx065 28475664

[36] Engel GL. The need for a new medical model: a challenge for biomedicine. Science. (1977) 196:129–36. 10.1126/science.847460 847460

[37] Engel GL. The clinical application of the biopsychosocial model. Am J Psychiatry. (1980) 137:535–44. 10.1176/ajp.137.5.535 7369396

[38] van Uexküll T Pauli HG The mind-body problem in medicine. Adv J Institute Adv Health. (1986) 3:158–74.

[39] McWhinney IR. An acquaintance with particulars. Fam Med. (1989) 21:296–8. 2753257

[40] Bircher J. Towards a dynamic definition of health and disease. Med Health Care Philos. (2005) 8:335–41. 10.1007/s11019-005-0538-y 16283496

[41] Bircher H Kuruvilla S. Defining health by addressing individual, social, and environmental determinants: new opportunities for health care and public health. J Public Health Pol. (2014) 35:363–86. 10.1057/jphp.2014.19 24943659 PMC4119253

[42] Huber M Knottnerus JA Green L van de Horst H Jadad AR Kromhout D. How should we define health? Br Med J. (2011) 343:d4163. 10.1136/bmj.d4163 21791490

[43] Schrödinger E What Is Life? The Physical Aspect of the Living Cell. Cambridge: Cambridge University Press (1944).

[44] Macklem PT. Emergent phenomena and the secrets of life. J Appl Physiol. (2008) 104:1844–6. 10.1152/japplphysiol.00942.2007 18202170

[45] Macklem PT Seely A. Towards a definition of life. Perspect Biol Med. (2010) 53:330–40. 10.1353/pbm.0.0167 20639603

[46] Picard M McEwen BS Epel E Sandi C. An energetic view of stress: focus on mitochondria. Front Neuroendocrinol. (2018) 49:72–85. 10.1016/j.yfrne.2018.01.001 29339091 PMC5964020

[47] Australian Bureau of Statistics Australian Health Survey: Updated Results, 2011-12, Australian Bureau of Statistics. Canberra (2013).

[48] Australian Bureau of Statistics General Social Survey: Summary Results, Australia, 2014, Australian Bureau of Statistics. Canberra (2015).

[49] van den Akker M Buntinx F Knottnerus JA Comorbidity or multimorbidity. Eur J General Pract. (1996) 2:65–70. 10.3109/13814789609162146

[50] Goh K-I Cusick ME Valle D Childs B Vidal M Barabási A-L. The human disease network. Proc Natl Acad Sci. (2007) 104:8685–90. 10.1073/pnas.0701361104 17502601 PMC1885563

[51] Tracey KJ. Physiology and immunology of the cholinergic antiinflammatory pathway. J Clin Invest. (2007) 117:289–96. 10.1172/JCI30555 17273548 PMC1783813

[52] Glaser R Kiecolt-Glaser JK. Stress-induced immune dysfunction: Implications for health. Nat Rev Immunol. (2005) 5:243–51. 10.1038/nri1571 15738954

[53] Cole SW. Social regulation of human gene expression: mechanisms and implications for public health. Am J Public Health. (2013) 103:S84–92. 10.2105/AJPH.2012.301183 23927506 PMC3786751

[54] Wallace DC. A mitochondrial bioenergetic etiology of disease. J Clin Invest. (2013) 123:1405–12. 10.1172/JCI61398 23543062 PMC3614529

[55] Picard M Juster R-P McEwen BS. Mitochondrial allostatic load puts the 'gluc' back in glucocorticoids. Nat Rev Endocrinol. (2014) 10:303–10. 10.1038/nrendo.2014.22 24663223

[56] Heng H Bremer S Stevens J Horne S Liu G Abdallah B. Chromosomal instability (CIN): what it is and why it is crucial to cancer evolution. Cancer Metastasis Rev. (2013) 32:325–40. 10.1007/s10555-013-9427-7 23605440

[57] Heng HHQ. The genome-centric concept: resynthesis of evolutionary theory. Bioessays. (2009) 31:512–25. 10.1002/bies.200800182 19334004

[58] Kaur H Carvalho J Looso M Singh P Chennupati R Preussner J. Single-cell profiling reveals heterogeneity and functional patterning of GPCR expression in the vascular system. Nat Commun. (2017) 8:15700. 10.1038/ncomms15700 28621310 PMC5481776

[59] Luo C Keown CL Kurihara L Zhou J He Y Li J. Single-cell methylomes identify neuronal subtypes and regulatory elements in mammalian cortex. Science. (2017) 357:600–4. 10.1126/science.aan3351 28798132 PMC5570439

[60] Heng HH Debating Cancer: The Paradox in Cancer Research. Singapore: World Scientific Publishing (2016).

[61] Barabási A-L. Network medicine — from obesity to the “diseasome”. N Engl J Med. (2007) 357:404–7. 10.1056/NEJMe078114 17652657

[62] Selye HA A syndrome produced by diverse nocuous agents. Nat Biotechnol. (1936) 138:32. 10.1176/jnp.10.2.230a 9722327

[63] Porges SW. The polyvagal theory: new insights into adaptive reactions of the autonomic nervous system. Cleve Clin J Med. (2009) 76:S86–90. 10.3949/ccjm.76.s2.17 19376991 PMC3108032

[64] Kolacz J Porges SW Chronic diffuse pain and functional gastrointestinal disorders after traumatic stress: pathophysiology through a polyvagal perspective. Front Med. (2018) 5:145 10.3389/fmed.2018.00145 PMC5990612 29904631

[65] Karatoreos IN McEwen BS. Annual research review: the neurobiology and physiology of resilience and adaptation across the life course. J Child Psychol Psychiatry. (2013) 54:337–47. 10.1111/jcpp.12054 23517425

[66] Lazarus RS Folkman S Stress, Appraisal, and Coping. New York, NY: Springer (1984).

[67] Antonovsky A Health, Stress and Coping. San Francisco, CA: Jossey-Bass (1979).

[68] Antonovsky A. Complexity, conflict, chaos, coherence, coercion and civility. Soc Sci Med. (1993) 37:969–74. 10.1016/0277-9536(93)90427-6 8235741

[69] Rohleder NP. Stimulation of systemic low-grade inflammation by psychosocial stress. Psychosom Med. (2014) 76:181–9. 10.1097/PSY.0000000000000049 24608036

[70] Wolf JM Rohleder N Bierhaus A Nawroth PP Kirschbaum C. Determinants of the NF-κB response to acute psychosocial stress in humans. Brain Behav Immun. (2009) 23:742–9. 10.1016/j.bbi.2008.09.009 18848620

[71] Bierhaus A Wolf J Andrassy M Rohleder N Humpert PM Petrov D. A mechanism converting psychosocial stress into mononuclear cell activation. Proc Natl Acad Sci USA. (2003) 100:1920–5. 10.1073/pnas.0438019100 12578963 PMC149934

[72] Rohleder N Marin TJ Ma R Miller GE. Biologic cost of caring for a cancer patient: dysregulation of pro- and anti-inflammatory signaling pathways. J Clin Oncol. (2009) 27:2909–15. 10.1200/JCO.2008.18.7435 19433690

[73] Cohen S Janicki-Deverts D Doyle WJ Miller GE Frank E Rabin BS. Chronic stress, glucocorticoid receptor resistance, inflammation, and disease risk. Proc Natl Acad Sci USA. (2012) 109:5995–9. 10.1073/pnas.1118355109 22474371 PMC3341031

[74] Picard M McEwen BS. Psychological stress and mitochondria: a conceptual framework. Psychosom Med. (2018) 80:126–40. 10.1097/PSY.0000000000000544 29389735 PMC5901651

[75] Picard M McEwen BS. Mitochondria impact brain function and cognition. Proc Natl Acad Sci. (2014) 111:7–8. 10.1073/pnas.1321881111 24367081 PMC3890847

[76] Naik E Dixit VM. Mitochondrial reactive oxygen species drive proinflammatory cytokine production. J Exp Med. (2011) 208:417–20. 10.1084/jem.20110367 21357740 PMC3058577

[77] Chinnery PF Howell N Lightowlers RN Turnbull DM. Molecular pathology of MELAS and MERRF. The relationship between mutation load and clinical phenotypes. Brain Cogn. (1997) 120:1713–21. 10.1093/brain/120.10.1713 9365365

[78] Maeda K. Clinical Phenotype and segregation of mitochondrial 3243A>G mutation in 2 pairs of monozygotic twins. JAMA Neurol. (2016) 73:990–3. 10.1001/jamaneurol.2016.0886 27323007

[79] Picard M McEwen BS. Psychological stress and mitochondria: a systematic review. Psychosom Med. (2018) 80:141–53. 10.1097/PSY.0000000000000545 29389736 PMC5901654

[80] Picard M McManus MJ Gray JD Nasca C Moffat C Kopinski PK. Mitochondrial functions modulate neuroendocrine, metabolic, inflammatory, and transcriptional responses to acute psychological stress. Proc Natl Acad Sci. (2015) 112:E6614–23. 10.1073/pnas.1515733112 26627253 PMC4672794

[81] McEwen BS. Neurobiological and Systemic Effects of Chronic Stress. Thousand Oaks, CA: Chronic stress (2017). 10.1177/2470547017692328 PMC5573220 28856337

[82] McEwen BS. Allostasis and the epigenetics of brain and body health over the life course: the brain on stress. JAMA Psychiatry. (2017) 74:551–2. 10.1001/jamapsychiatry.2017.0270 28445556

[83] Robertson T Beveridge G Bromley C. Allostatic load as a predictor of all-cause and cause-specific mortality in the general population: evidence from the Scottish Health Survey. PLOS ONE. (2017) 12:e0183297. 10.1371/journal.pone.0183297 28813505 PMC5559080

[84] Remick DG Friedland JS editors. Cytokines in Health and Disease. New York, NY: M. Dekker (1997).

[85] Petrovsky N Harrison LC. The chronobiology of human cytokine production. Int Rev Immunol. (1998) 16:635–49. 10.3109/08830189809043012 9646180

[86] Sapolsky R Rivier C Yamamoto G. Interleukin-1 stimulates the secretion of hypothalmic corticotropin-releasing factor. Science. (1987) 238:522–6. 10.1126/science.2821621 2821621

[87] Dantzer R. Innate immunity at the forefront of psychoneuroimmunology. Brain Behav Immun. (2004) 18:1–6. 10.1016/j.bbi.2003.09.008 14651940

[88] Dantzer R Kelley KW. Twenty years of research on cytokine-induced sickness behavior. Brain Behav Immun. (2007) 21:153–60. 10.1016/j.bbi.2006.09.006 17088043 PMC1850954

[89] Watkins LR Maier SF. Implications of immune-to-brain communication for sickness and pain. Proc. Natl Acad. Sci. USA. (1999) 96:7710–3. 10.1073/pnas.96.14.7710 10393885 PMC33606

[90] Banks WA Erickson MA. The blood–brain barrier and immune function and dysfunction. Neurobiol Dis. (2010) 37:26–32. 10.1016/j.nbd.2009.07.031 19664708

[91] Yang Y Nishikawa T Motter AE Small vulnerable sets determine large network cascades in power grids. Science. (2017) 2017:358 10.1126/science.aan3184 29146778

[92] Blane D Kelly-Irving M Errico A Bartley M Montgomery S Social-biological transitions: how does the social become biological? Life Course Stud. (2013) 4:11 10.14301/llcs.v4i2.236

[93] Slavich GM Cole SW. The emerging field of human social genomics. Clin Psychol Sci. (2013) 1:331–48. 10.1177/2167702613478594 23853742 PMC3707393

[94] Cole SW. Human social genomics. PLoS Genet. (2014) 10:e1004601. 10.1371/journal.pgen.1004601 25166010 PMC4148225

[95] Marmot M. The influence of income on health: views of an epidemiologist. Health Aff. (2002) 21:31–46. 10.1377/hlthaff.21.2.31 11900185

[96] Commission on Social Determinants of Health Closing the Gap in a Generation: Health Equity Through Action on the Social Determinants of Health. Final Report of the Commission on Social Determinants of Health, World Health Organization, Geneva (2008).

[97] Institute of Medicine Committee on Health and Behavior, Health and Behavior: The Interplay of Biological, Behavioral, and Societal Influences. Washington, DC: National Academy Press (2001). 20669491

[98] Frasca D Blomberg BB Paganelli R Aging, obesity, and inflammatory age-related diseases. Front Immunol. (2017) 2017:8 10.3389/fimmu.2017.01745 PMC5725402 29270179

[99] McManus J Constable M Bunten A Chadborn T Improving People's Health: Applying Behavioural and Social Sciences to Improve Population Health and Wellbeing in England. London: Public Health England (2008).

[100] Idler EL Benyamini Y. Self-rated health and mortality: a review of twenty-seven community studies. J Health Soc Behav. (1997) 38:21–37. 10.2307/2955359 9097506

[101] Jylhä M. What is self-rated health and why does it predict mortality? Towards a unified conceptual model. Soc Sci Med. (2009) 69:307–16. 10.1016/j.socscimed.2009.05.013 19520474

[102] Benyamini Y. Why does self-rated health predict mortality? An update on current knowledge and a research agenda for psychologists. Psychol Health. (2011) 26:1407–13. 10.1080/08870446.2011.621703 22111660

[103] Latham K Peek CW. Self-rated health and morbidity onset among late midlife U.S. adults. J Gerontol Ser B. (2013) 68:107–16. 10.1093/geronb/gbs104 23197340 PMC3605944

[104] Bravi A Green G Longtin A Seely JE. Monitoring and identification of sepsis development through a composite measure of heart rate variability. PLoS ONE. (2012) 7:e45666. 10.1371/journal.pone.0045666 23029171 PMC3446945

[105] Jarczok MN Kleber ME Koenig J Loerbroks A Herr RM Hoffmann K. Investigating the associations of self-rated health: heart rate variability is more strongly associated than inflammatory and other frequently used biomarkers in a cross sectional occupational sample. PLOS ONE. (2015) 10:e0117196. 10.1371/journal.pone.0117196 25693164 PMC4333766

[106] Mayo Clinic Our Shared Committment - Mayo Clinic 2007 Annual Report (2007).

[107] Sturmberg JP O'Halloran DM Martin CM. People at the centre of complex adaptive health systems reform. Med J Aust. (2010) 193:474–8. 10.5694/j.1326-5377.2010.tb04004.x 20955127

[108] Sturmberg JP O'Halloran DM Martin CM Health care reform - the need for a complex adaptive systems approach. In: Sturmberg JP Martin CM editors. Handbook of Systems and Complexity in Health. New York, NY: Springer (2013). p. 827–53.

[109] Seltman KD Berry LL Mayo clinic: making complex healthcare simpler. In: Sturmberg JP Martin CM editors. Handbook of Systems and Complexity in Health. New York, NY: Springer (2013). p. 685–96.

[110] Sturmberg JP Health System Redesign. How to Make Health Care Person-Centered, Equitable, and Sustainable. Cham: Springer (2018).

[111] Antonovsky A Unraveling the Mystery of Health - How People Manage Stress and Stay Well. San Francisco, CA: Jossey-Bass Publishers (1987).

[112] Kaminski M Popping the Question. (2015). Available online at: https://pulsevoices.org/index.php/archive/stories/451-popping-the-question

[113] Stein AD Lumey LH. The relationship between maternal and offspring birth weights after maternal prenatal famine exposure: the dutch famine birth cohort study. Hum Biol. (2000) 72:641–54. 10.1385/1-59259-216-3:101 11048791

[114] Yehuda R Engel SM Brand SR Seckl J Marcus SM Berkowitz GS. Transgenerational effects of posttraumatic stress disorder in babies of mothers exposed to the world trade center attacks during pregnancy. J Clin Endocrinol Metabol. (2005) 90:4115–8. 10.1210/jc.2005-0550 15870120

[115] Yehuda R Teicher MH Seckl JR Grossman RA Morris A Bierer LM. Parental posttraumatic stress disorder as a vulnerability factor for low cortisol trait in offspring of holocaust survivors. Arch. Gen. Psychiatry. (2007) 64:1040–8. 10.1001/archpsyc.64.9.1040 17768269

[116] Lindeboom M Portrait F van den Berg GJ. Long-run effects on longevity of a nutritional shock early in life: the Dutch Potato famine of 1846–1847. J Health Econ. (2010) 29:617–29. 10.1016/j.jhealeco.2010.06.001 20663577

[117] DeHaven MJ Gimpel NE. Reaching out to those in need: the case for community health science. J Am Board Family Med. (2007) 20:527–32. 10.3122/jabfm.2007.06.060148 17954859

[118] Horowitz CR Robinson M Seifer S. Community-based participatory research from the margin to the mainstream. Are Researchers Prepared? (2009) 119:2633–42. 10.1161/CIRCULATIONAHA.107.729863 19451365 PMC2796448

[119] Woolf SH. The meaning of translational research and why it matters. JAMA. (2008) 299:211–3. 10.1001/jama.2007.26 18182604

[120] J.Ioannidis PA Why most clinical research is not useful. PLOS Med. (2016) 13:e1002049 10.1371/journal.pmed.1002049 27328301 PMC4915619

[121] Starfield B.Politics, primary healthcare and health: was Virchow right? J Epidemiol Commun Health. (2011) 65:653–5. 10.1136/jech.2009.102780 21727176

[122] Stevens JB Abdallah BY Liu G Ye CJ Horne SD Wang G. Diverse system stresses: common mechanisms of chromosome fragmentation. Cell Death and Dis. (2011) 2:e178. 10.1038/cddis.2011.60 21716293 PMC3169002

[123] Thayer JF Åhs F Fredrikson M Sollers JJ Wager TD. A meta-analysis of heart rate variability and neuroimaging studies: implications for heart rate variability as a marker of stress and health. Neurosci Biobehav Rev. (2012) 36:747–56. 10.1016/j.neubiorev.2011.11.009 22178086

[124] Sturmberg JP Bennett JM Picard M Seely JE. The trajectory of life. Decreasing physiological network complexity through changing fractal patterns. Front Physiol. (2015) 6:169. 10.3389/fphys.2015.00169 26082722 PMC4451341

[125] Ernst G. Heart-rate variability—more than heart beats? Front Public Health. (2017) 5:240. 10.3389/fpubh.2017.00240 28955705 PMC5600971

[126] Geller SM Porges SW Therapeutic presence: neurophysiological mechanisms mediating feeling safe in therapeutic relationships. J Psychother Integr. (2014) 24:178–92. 10.1037/a0037511

[127] Boyd CM Darer J Boult C Fried LP Boult L Wu AW. Clinical practice guidelines and quality of care for older patients with multiple comorbid diseases: implications for pay for performance. JAMA. (2005) 294:716–24. 10.1001/jama.294.6.716 16091574

[128] Spira JL Drury R Resilience Bolstering. In: Figley CR editor. Encyclopedia of Trauma. An Interdisciplinary Guide. Los Angeles, CA: Sage Reference (2012). p. 556–61. 10.4135/9781452218595.n188

[129] Southwick SM Charney DS. The science of resilience: implications for the prevention and treatment of depression. Science. (2012) 338:79–82. 10.1126/science.1222942 23042887

